# Glioblastoma multiforme: Diagnosis, treatment, and invasion

**DOI:** 10.7555/JBR.36.20220156

**Published:** 2022-10-28

**Authors:** Jiawei Li, Lili Feng, Yingmei Lu

**Affiliations:** 1 Department of Physiology, School of Basic Medical Sciences, Nanjing Medical University, Nanjing, Jiangsu 211166, China; 2 The First Clinical Medical College of Nanjing Medical University, Nanjing, Jiangsu 211166, China; 3 Key Laboratory of Cardiovascular & Cerebrovascular Medicine, Drug Target and Drug Discovery Center, School of Pharmacy, Nanjing Medical University, Nanjing, Jiangsu 211166, China

**Keywords:** glioblastoma multiforme, diagnosis, treatment, patterns of invasion, invasion mechanism

## Abstract

Glioblastoma multiforme (GBM) is an essentially incurable brain tumor, which has been explored for approximately a century. Nowadays, surgical resection, chemotherapy, and radiation therapy are still the standardized therapeutic options. However, due to the intrinsic invasion and metastasis features and the resistance to chemotherapy, the survival rate of glioblastoma patients remains unsatisfactory. To improve the current situation, much more research is needed to provide comprehensive knowledge of GBM. In this review, we summarize the latest updates on GBM treatment and invasion. Firstly, we review the traditional and emerging therapies that have been used for GBM treatment. Given the limited efficiency of these therapies, we further discuss the role of invasion in GBM recurrence and progression, and present current research progress on the mode and mechanisms of GBM invasion.

## Introduction

Glioblastoma multiforme (GBM) is the most aggressive malignant brain tumor with a high incidence rate and a low survival rate. It accounts for approximately 14.7% of all central nervous system tumors (CNSTs), and 56.5% of gliomas^[1]^. The overall incidence rate in the United States is 4.23%, while Asians/Pacific islanders have a relatively low incidence rate of 2.00%^[2]^. With a bleak prognosis due to the high aggression and recurrence rate, the median survival time is 12 months in all GBM cases, and the average 2-year and 5-year survival rates were 21.3% and 13.8% respectively^[3]^. In China, the burden of CNSTs is nonnegligible with a large number of affected individuals, and the overall CNST incident cases have increased by 106.52% within the past three decades^[4]^.

Primary GBM, accounting for 94.7% of GBM, has a mean age at occurrence between 59 and 62, while a secondary GBM, which is rarer, occurs at a relatively young age. The two types of glioblastomas both originate from glial progenitor cells or neural stem cells, but the secondary GBM derives from astrocytoma^[5]^. Normally, patients are treated with combined therapies, including chemotherapy, surgery, or radiotherapy, aiming to ameliorate the frustrating survival rate. Nevertheless, the intricate infiltration nature and heterogeneity of GBM hinder the complete eradication of the tumor, thus contributing to a high recurrence rate^[6]^.

GBM invasion induced by the diffuse infiltration and invasion margin has caught the attention from clinic doctors, pathologists, and pharmacologists for a long time. GBM cells invade following the Scherer structure, which was named after a German pathologist who defined it. These cells infiltrate along existing brain structures like brain parenchyma, blood vessels, white matter tracts, and subpial spaces. Despite their preference for the white matter, they migrate fastest alongside blood vessels, mostly unidirectionally, and in a helical movement^[7]^.

Among various subtypes of glioblastomas, the mesenchymal subtype is the most liable to invade. Other phenotypes may also transit into the mesenchymal subtype through the mesenchymal transition^[8]^. Various pathways have been shown to contribute to GBM invasion. In addition, the microenvironment also provides structural support for GBM cells and may act as a guiding scaffold in the process of invasion.

In this review, we take a glance at different methods of treatment and diagnosis, including the common practice and some future prospects. When further exploring the poor treatment outcomes and prognosis, we found the key contribution of invasion. So, we discuss the current knowledge of the mode and dynamics of invasion, and depict a holistic picture of mechanisms whereby GBM cells invade. Based on these findings, we hope to provide more comprehensive knowledge about GBM for researchers to develop more effective treatment options.

## Diagnosis

**Figure 1:**
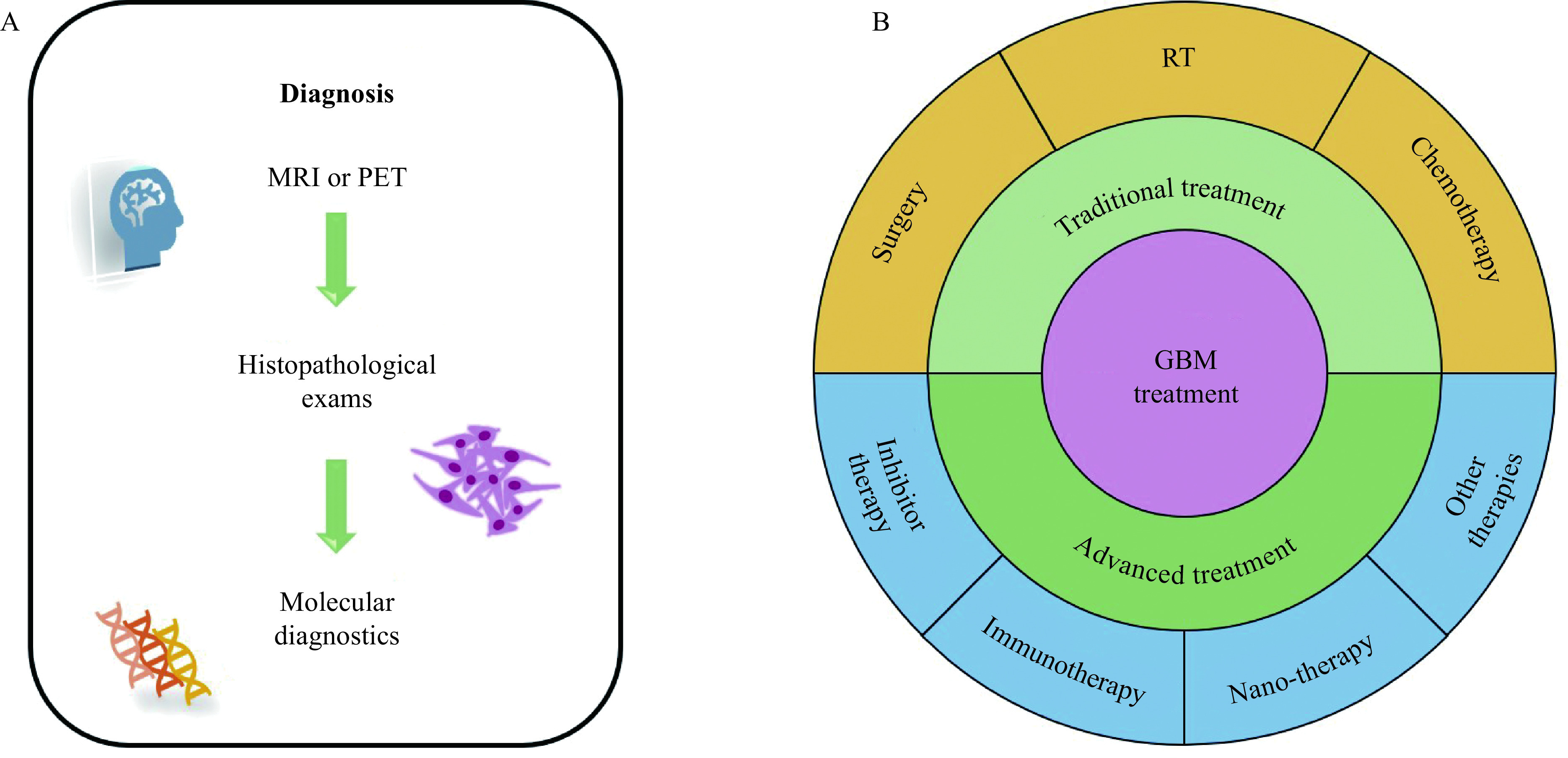
Glioblastoma multiforme diagnosis and treatment.

Techniques employed in the diagnosis of GBM comprise invasive and non-invasive ones, as shown in ***Fig. 1A***. Contrast-enhanced magnetic resonance imaging (MRI) is the most widely used non-invasive technique^[9]^. For highly specific imaging, positron emission tomography (PET) can be considered, which is recommended for the diagnosis of level Ⅲ/Ⅳ glioblastoma. Another innovative option is "immunotargeted imaging" in which the high target-specific antibodies combine with the given tumor cell surface target, and PET is subsequently used for imaging. This process brings the real-time monitoring to the reality. The collaboration of immunohistochemistry and PET (immuno-PET) is thus called a "virtual biopsy"^[10]^.

For a definitive diagnosis, histopathological examinations are necessary, which requires tumor resection. If metastatic GBM is suspected, fine needle aspiration cytology is relatively more reliable, especially in the study of extra-cranial metastases to the parotid gland^[11]^. With the discovery of more circulating biomarkers, the liquid biopsy may become a trending auxiliary examination in the future. Serum, plasma, and cerebrospinal fluid can be sampled and analyzed for biomarkers like ctDNA, miRNA, proteins, and exosomes. Tumor cells disseminated into the fluid might also be found in a liquid biopsy^[12]^.

To predict the invasiveness and the prognosis of GBM, testing for the mutation status of biomarkers is required. One of the most important biomarkers is isocitrate dehydrogenase 1 and 2 (*IDH1*/*IDH2*), whose mutations have been used for GBM classification and prognosis prediction^[9]^. Chromosome 1p and 19q codeletion, mutations in * ATRX*, telomerase reverse transcriptase (*TERT*) promoter, tumor protein p53 (*TP53*), and B-raf proto-oncogene as well as serine/threonine kinase (*BRAF*) V600E mutation are also supportive for GBM diagnosis^[13]^. Moreover, O^6^-methylguanine-DNA methyltransferase (MGMT) promoter methylation is employed to guide GBM treatment^[14]^. Other crucial mutations occurring in phosphatase and tensin homolog (PTEN), and variants of histone 3, the epidermal growth factor receptor (EGFR), Ki-67, and glial fibrillary acidic protein (GFAP) are reported to accelerate the progression of GBM. In addition, their mutational landscape potentially contributes to GBM subclassification and is of prognostic value, yet none of which are used as molecular biomarkers in clinical practice^[15–19]^. To find the much more specific biomarkers for GBM diagnosis apart from *IDH*, 1p/19q, and MGMT, further investigations are needed.

## Treatment

Traditional treatments are the standard approach used for newly diagnosed GBM patients. Surgery is the first choice of the standardized GBM therapies. Maximal safe surgical resection is recommended for cases that are not suitable for total eradication, like invasive or metastatic GBM^[9]^. Depending on the extent of tumor resection, surgical options are divided into four types, including gross total resection (GTR), subtotal resection (SR), partial resection (PR), and biopsy^[20]^. A second or a third surgery might be needed in some cases, but the outcome is unsatisfactory. Patients who have received surgeries may suffer from seizures and focal neurological deficits. Headache, nausea, stupor, and unconsciousness due to intracranial hypertension are also the common side effects^[21]^.

As an alternative therapy, RT has shown clear advantages due to its non-invasiveness. 50 to 60 Gy is suitable for the most cases, which can eliminate microscopic lesions after tumor resection^[22]^, and with the application of imaging techniques, including MRI, the radiation can be limited effectively to a local extent^[23]^. The 3D RT with a portal imaging is the recommended technique for GBM patients. For those under the age of 70 or in good general health, RT beginning within four to six weeks after surgery or even earlier, in combination with chemotherapy, is the ideal choice. An accelerated hypofractionated RT regimen is applicable to patients over the age of 70 years and those in poor general health^[24]^. However, tumor relapse is still inevitable due to tumor cell-intrinsic or tumor microenvironment-mediated resistance to RT^[25]^.

In addition to surgical treatment and RT, the standard postoperative care also include chemotherapy. Only three chemotherapeutic agents are approved by the Food and Drug Administration (FDA) now. The first class is nitrosoureas, including carmustine and lomustine, used for GBM chemotherapy over 40 years ago^[26]^. However, due to liver and kidney toxicities, they are mostly abandoned in treatment^[27]^. Implantable carmustine pumps are still used for the local delivery of medicine in the resection cavity to improve the survival rate of both the newly diagnosed and recurrent GBM^[28]^. The second approved agent is temozolomide (TMZ), which is commonly used in treating newly diagnosed malignant brain tumors. It can cross the blood-brain barrier quickly, disrupt DNA replication, and cause modification and cross-linking of DNA, which mostly results in apoptosis of rapidly dividing cells located in the brain^[29]^. The biggest problem of TMZ is that patients were susceptible to resistance. One of the main contributors to TMZ resistance is MGMT, which repairs TMZ-induced DNA alkylation. Base excision repair and autophagy are other suspected contributors^[30]^. New methods have been adopted to overcome TMZ resistance. As the first anti-angiogenic agent widely used to treat various tumors, bevacizumab has been approved to treat GBM in combination with TMZ and RT^[31]^, which can reduce glucocorticoid requirements to lower the risk of morbidity and other side effects caused by glucocorticoids. The use of bevacizumab is now the first-line treatment for a relapsed or progressing GBM, slightly increasing patients' progression-free survival^[32]^.

Despite the standard therapeutic strategy used in GBM, the survival rate is still frustrating. Novel approaches are currently being explored, some of which have been approved for clinical trials. Inhibitor therapy, one of the high-profile molecular targeting treatments, typically targets a particular kinase or a group of kinases that are excessively activated in malignant tissues. Rearrangement, amplification, and fusion of receptor tyrosine kinase (RTK) are observed in GBM, making them the appealing targets for advanced treatment^[33]^. The EGFR inhibitor Afatinib, which is approved by the FDA to treat non-small-cell lung cancer, increases the overall survival of GBM patients when combined with TMZ^[34]^. Studies and trials for other EGFR inhibitors, including erlotinib and gefitinib, are also being conducted^[35–36]^. Besides RTK inhibitors, studies and trials for adenosine diphosphate (ADP) ribose polymerase inhibitors, myeloid cell leukemia-1, and topoisomerase are underway as well^[33]^.

The inbuilt immune system is the defense wall and strong weapon against pathogens and cancer cells. However, the immune system's ability to eliminate abnormal cells was suppressed under the tumor microenvironment. Immunotherapy has been proved to be effective in non-small cell lung cancer and quite a few other cancers via manipulating the related immune cells to rescue their ability to attack cancer cells. However, as immunologically cold tumors, gliomas are relatively insensitive to immunotherapy. The main forms of GBM immunotherapy under investigation include peptide vaccines, dendritic cell vaccines, chimeric T-cell receptors, checkpoint inhibitors, and oncolytic virotherapy^[37]^. Checkpoint inhibitors, such as anti-cytotoxic T-lymphocyte-associated protein 4 (CTLA-4) and anti-programmed cell death protein 1 (PD-1) drugs, appear to produce amphibolous outcomes. These may pioneer GBM immunotherapy through the conduct of numerous preclinical studies and clinical trials^[38]^.

The nano-therapy refers to the utilization of nanoparticulate anti-GBM drugs. Gold nanosphere, gold nanorods, carbon nanotubes, nanogels, polymeric nanoparticles, polymeric micelles, and liposomes are the main carriers of nanoparticulate anti-GBM drugs currently under investigation^[33]^. The unique characteristics of these novel materials result in easier diffusion through the blood-brain barrier, enhanced permeability and retention effect, and a homogenous distribution within the tumor. With the application of these strategies, nano-formulated drugs, such as erlotinib, can be delivered through liposomal nanoparticles specifically to tumor cells^[39–40]^.

There are also some other new therapeutic strategies attempting to improve the prognosis of GBM. Tumor treating fields (TTF) therapy, also known as alternating electric field therapy, is a kind of non-invasive GBM treatment, which delivers low-intensity (1 to 3 V/cm), intermediate-frequency (100 to 300 kHz), alternating electric fields transcutaneously. The electric fields exert biophysical force on charged and polarizable molecules known as dipoles. TTF therapy has antimitotic effects and can interfere with DNA repair, thus potentially suppressing TMZ resistance mentioned earlier. Prevention of the inhibitory effects of the phosphatidylinositol 3-kinase (PI3K)/AKT/mammalian target of rapamycin (mTOR) signaling pathway on autophagy is another possible function of the TTF therapy in GBM treatment, because the PI3K pathway is closely related to GBM invasion. Antitumor immunity and anti-migratory through increasing cell membrane permeability are important anti-tumor mechanisms of the TTF therapy as well^[41]^.

Laser interstitial thermal therapy (LITT) is a neurosurgical technique utilizing thermal energy. Directed by the stereotaxic device, an optical fiber generates heat in the center of the tumor and burns tumor cells. The procedure only leaves a small hole on the skull and barely affects healthy tissues surrounding the tumor^[33]^. Prolonged survival of newly diagnosed GBM patients after LITT has been demonstrated^[42]^. For patients with unresectable tumors, the application of LITT is promising.

Stem cell-based therapy is the union of at least five types of cancer stem cell (CSC) targeted therapy, including chemoradiotherapy with radiosensitizers and chemotherapeutics, and immunotherapy mentioned above^[43]^. It is mainly based on the stem cell theory, in which CSCs in the brain tissue originate from stem cells with accumulated mutations, and the uncontrolled migration of CSCs finally causes tumorigenesis. A big merit of the stem cell-based therapy is the minimal side effects, but a multitude of issues remain to be addressed, for example, biosafety^[44]^.

Currently, systematic treatment of GBM includes surgery, radiation therapy (RT), and chemotherapy. Advanced therapies vary from research hotspots, such as immunotherapy, nano-therapy, and inhibitor therapy, to TTF therapy and LITT (***Fig. 1B***). A traditional therapy is still the preferred choice when patients are first diagnosed with glioblastoma. Patients who receive chemotherapy or RT besides tumor resection have a better prognosis than those who do not^[45]^. Despite the application of improved protocols, *i.e.*, the Stupp protocol, in which the doses of chemotherapeutic agents maintain at a low level, a recurrence is still almost a certainty due to drug or RT resistance. For progressing or recurrent GBM, additional advanced therapy is urgently needed. It is predictable that molecular targeted therapy and immune therapy will be added to the option list of GBM treatment in the future with the development of precision medicine. The combination of different therapies is also a future trend with more evidence supportive of its efficacy. The TTF therapy is already recommended in China, while clinical trials are needed for the addition of LITT to the standard treatment. To retard the ominous process of recurrence and increase survival, improvements in GBM treatment should be put on the top of the agenda.

## The patterns and dynamics of glioblastoma multiforme invasion

GBM cell invasion follows specific patterns. Although it can be attributed to the intrinsic genetic features of tumor cells or their interactions with the microenvironment, generally, there are several crucial signaling pathways supporting tumor cells cooperatively, including the p53 and the RTK pathways, as well as various pathways related to epithelial-mesenchymal transition (EMT). Growth factors, chemokines, and integrins are also involved in the process of metastasis and invasion^[8]^.

In the 1930s, German pathologist Hans Joachim Scherer first defined the routes for GBM invasion, which is the prelude to systematic research into the patterns and dynamics of GBM invasion. According to his observation, gliomas are localized in the white matter at first and can migrate along white matter structures, blood vessels, and ependymal surfaces, which is later referred to as the Scherer structure^[46]^. Tamura *et al* demonstrated that glioma stem cells (GSCs) possess a tendency to migrate towards the anterior corpus callosum from all directions, while the cortical area seems less likely to be the destination. This might be due to the complex and tight connections of neurons in these areas. These observations further proved Scherer's anticipation that GBM cells progress along paths with the least obstruction^[47]^.

GBM invasion starts from the migration of tumor cells located at the border, and usually, there is a leader cell, followed by other invading cells. An "invasive margin", defined by Alieva *et al*, is the protruding multicellular groups originating at the interphase between the tumor and the brain parenchyma. With the highest proportion of invading cells that move in a directed way, the invasive margin configuration becomes the most aggressive type of border. Another aggressive border is the "diffuse margin", which means individual cell migration into the invasive area of the brain parenchyma. Invading cells here possess high a velocity but are less directed. The "well-defined border" refers to tumor margins without protrusions. Cells here are also dynamic, but in a spread-free way. In fact, even cells at the tumor core are not static, and migratory cells can be found in all areas of the tumor, indicating that mobility was one of the intrinsic characteristics of GBM cells. Due to the influence of the microenvironment, however, only specific cells at the aforementioned specific borders invade^[48]^.

## Signal pathways involved in invasion

**Figure 2:**
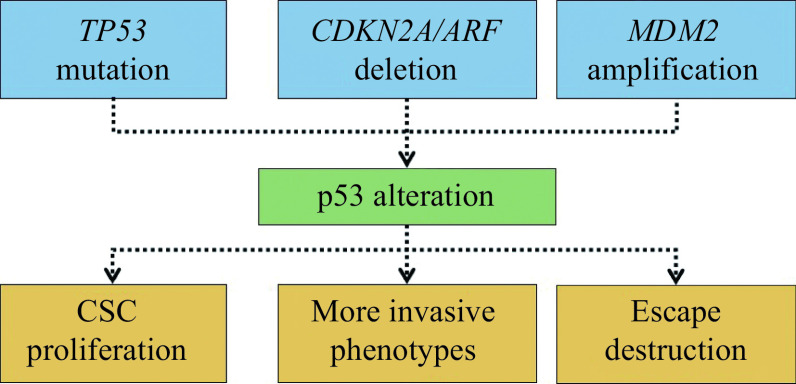
p53 pathway in glioblastoma multiforme invasion.

Referred to as the "Guardian of the Genome"^[49]^, p53 is a transcriptional regulator that prevents damaged cells from invasion by integrating stress signals and promoting cell cycle arrest^[50]^. Alteration of p53 found in 25% to 30% of primary GBM and 60% to 70% of secondary GBM, is the most common molecular abnormality in GBM^[51]^. Missense mutations of *TP53*, deletions of cyclin dependent kinase inhibitor 2A (*CDKN2A*/*ARF*), and amplification of mouse double minute 2 (*MDM2*) are ordinary mutations of the p53 pathway^[52]^ (***Fig. 2***). *TP53* plays a pivotal role in the proliferation of CSCs. With *TP53* mutations, CSCs are more likely to survive chemo-radiotherapy and thus leading to recurrence^[53]^. The *CDKN2A*/*ARF* locus is a frequently deregulated component of the p53 pathway. Retained ARF expression may generate more metastatic and invasive phenotypes of GBM^[54]^. MDM2, amplified in GBM, is an E3 protein ligase responsible for p53 degradation through a ubiquitin-dependent lysosome pathway^[55]^. Normally, p53 expression upregulates MDM2. However, mutant p53 cannot transactivate MDM2, so under the circumstances, it escapes destruction and causes various mutational effects, such as the overexpression of EGFR^[51]^. Inhibitors of MDM2/p53 interaction are under investigation and are promising in GBM treatment.

**Figure 3:**
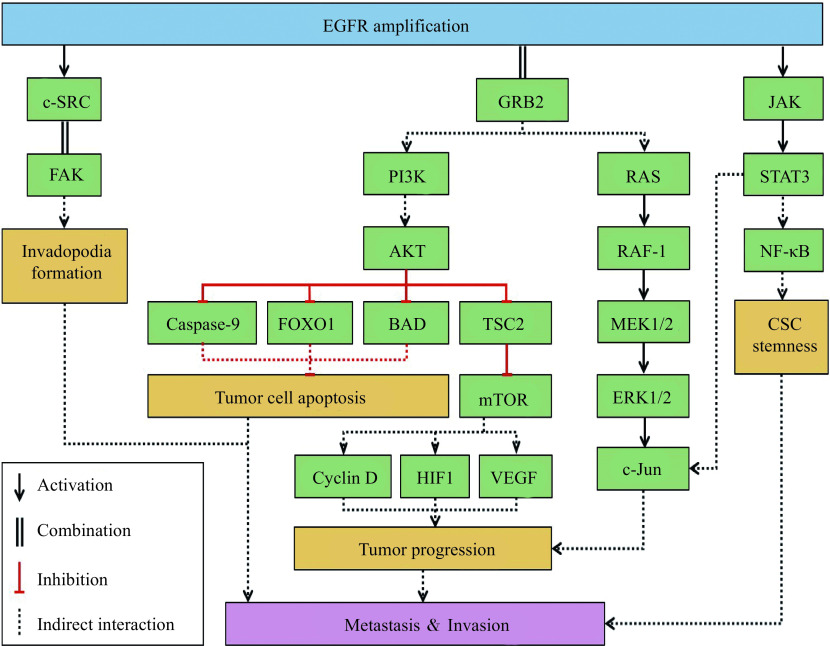
EGFR pathway in glioblastoma multiforme invasion.

RTKs are a kind of membrane-spanning proteins functioning as receptors of cytoplasmic signaling effectors after phosphorylation. Fifty-eight RTKs divided into 20 classes have been detected in humans, and the abnormality of some specific classes, for example, EGFR, insulin receptor (INSR), vascular endothelial growth factor receptor (VEGFR), and fibroblast growth factor receptor (FGFR), is closely related to GBM proliferation, invasion, and drug resistance, leading to a poor prognosis^[56]^. The EGFR family is a group of RTKs comprising EGFR, human epidermal growth factor receptor-2 (ErbB2/HER2), ErbB3/HER3, and ErbB4, of which EGFR is involved in the RAS/RAF/mitogen-activated protein kinase kinase (MEK)/extracellular signal-regulated kinase (ERK) pathway, the PI3K/AKT pathway, the Janus kinase (JAK)/signal transducer and activator of transcription (STAT) pathway, and the protein kinase C (PKC) pathway, and may play a primary role in the GBM onset, resistance to therapy, and recurrence^[56–57]^ (***Fig. 3***). Its amplification is commonly observed in GBM. Mutation, rearrangement, and altered splicing exist but are less frequent^[58]^. The RAS/RAF/MEK/ERK pathway is crucial to the regulation of cell proliferation, metabolism, survival, and apoptosis. Once transphosphorylated, EGFR binds to growth factor receptor bound protein 2 (GRB2) and recruits SHC, which next activates son of sevenless 1 (SOS1), a guanine nucleotide exchange factor, to induce RAS to exchange guanosine triphosphate (GTP) to guanosine diphosphate (GDP). Activated RAS leads to RAF-1 phosphorylation, allowing it to bind to MEK1/2. Phosphorylated by MEK1/2, ERK1/2, with over one hundred downstream cytoplasmic and nuclear substrates, induces a variety of biological responses^[59]^. In respect of GBM invasion, for example, as a result of EGFR amplification, ERK is upregulated and stabilizes YTH N^6^-methyladenosine RNA binding protein 2 (YTHDF2), thus promoting tumor propagation possibly due to repression of target genes expression, including L-xylulose reductase (*LXRA*) and HIVEP zinc finger 2 (*HIVEP2*)^[60]^.

Another important pathway involved in GBM invasion is the PI3K/AKT pathway. There are three classes of PI3K, among which Class Ⅰ PI3K is the downstream effector of EGFR. It can be recruited by the ErbB family or RAS and phosphorylates the membrane lipid phosphatidylinositol-4,5-bisphosphate (PIP_2_) to generate phosphatidylinositol-3,4,5-triphosphate (PIP_3_). PIP_3_ can activate AKT, which promotes cell proliferation by inhibiting caspase-9 and phosphorylating the pro-apoptotic BCL2 associated agonist of cell death (BAD). The indirect activation of mTOR due to the phosphorylation of TSC complex subunit 2 (TSC2), an inhibitor of mTOR, as a result of AKT activation, can cause increased synthesis of cyclin D1, hypoxia-inducible factor 1 (HIF1) and vascular endothelial growth factor (VEGF), which can promote tumor progression. AKT can also phosphorylate MDM2 and indirectly cause ubiquitination of p53^[59]^. By increasing glucose uptake and regulating glycolytic enzymes, constitutively-activated AKT stimulates the glycolysis of tumor cells^[61]^. However, to meet the bioenergetic needs, GBM does not possess a fixed metabolic phenotype, and instead, it can switch between the glycolytic phenotype and oxidative phenotype, which makes it easily resistant to metabolic therapy targeting AKT and continue to progress^[62]^. More research needs to be done to develop therapies that target the metabolic status of GBM cells.

EGFR also leads to the activation of the proto-oncogene c-SRC, the product of the avian tumor virus Rous sarcoma virus.^[59]^. Src family kinases bind to focal adhesion kinase (FAK) in integrin-mediated cell adhesion. The FAK/Src complex then regulates cellular functions, such as survival, proliferation, migration, and invasion, via downstream signaling pathways^[63]^. FAK overexpression also directly stimulates the formation of invadopodia and promotes their activity by controlling the localization of Src^[64]^. The JAK/STAT pathway is another important oncogenic pathway related to EGFR, which promotes GBM cell proliferation, angiogenesis, resistance to apoptosis, and immune escape through downstream targets, such as Bcl-xL, Bcl-2l1, Bcl-2, cyclin D1, and c-Myc. JAK is the abbreviation for Janus kinase, while STAT is short for signal transducer and activator of transcription. STAT is a family of seven transcription factors, including STAT1, STAT2, STAT3, STAT4, STAT-5a, STAT-5b, and STAT6. Their activation can be attributable to several signaling pathways, including cytokines, non-RTKs, and EGFR mentioned above. Of the seven members in the STAT family, STAT3 is the most widely discussed for tumor proliferation. It is associated with Notch signaling, and the JAK/STAT3 can be indirectly activated by transforming growth factor-β (TGF-β), which helps maintain the stemness of GSCs. TGF-β can bind to nuclear factor kappa-B (NF-κB), elevating the activity and the ability of GSC self-renewal^[65]^. The transcriptional regulation of GSCs largely contributes to Type 3 EMT, which will be discussed in the next part.

EMT is a very important process in organismal development, wound healing, and tissue fibrosis. During EMT, epithelial cells lose their junctions and apical-basal polarity, and the epithelial appearance changes into a spindle-shaped, mesenchymal morphology, which facilitates the invasion of the tumor with an increased cell motility^[66]^. The reversed process is called mesenchymal-epithelial transition (MET), which is needed to influence the metastatic competence at the site of recolonization^[67]^. EMT can be triggered by a range of stimuli, including hypoxia, alterations of metabolism, cytokines, growth factors, and anti-tumor drugs^[68]^. In particular, transcription factors zinc-finger E-box-binding (ZEB), the Snail family of zinc-finger transcription factors (SNAIL), SLUG (also a zinc-finger transcription factor), lymphoid enhancer factor (LEF), and the TWIST family have been proved to be related to EMT in GBM cell invasion via the repression of epithelial marker genes and activation of mesenchymal marker genes mediated by various signaling pathways. This leads to a decreased expression of E-cadherin, claudin, and occludins, which are essential to the detachment of tumor cells. To allow migration, proteases, such as matrix metalloproteinases (MMPs) and cathepsins, are produced to degrade the extracellular matrix (ECM) and form invadopodia^[69–72]^.

**Figure 4:**
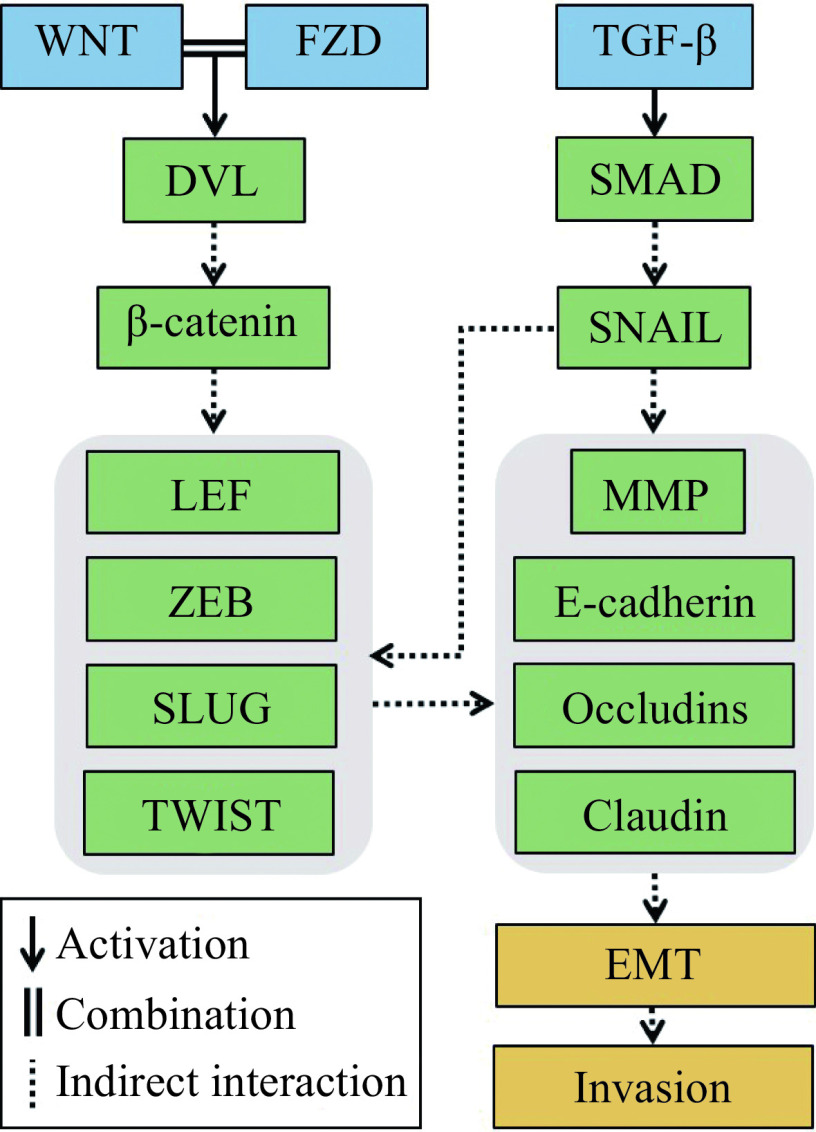
Pathways related to epithelial-mesenchymal transition.

One of the major signaling pathways inducing EMT is the TGF-β signaling pathway, which can be further divided into the mothers against decapentaplegic homologs (SMAD)-dependent signaling pathway (***Fig. 4***) and the SMAD-independent pathway. In the SMAD-dependent pathway, TGF-β phosphorylates SMAD2 and SMAD3, allowing them to bind to SMAD4 and translocate to the nucleus, which further induces the transcription of *ZEB*, *SNAI1* and *SANI2*, *LEF1*, and *TWIST*^[73]^. With the activation of RAS and PI3K by TGF-β, the SMAD-independent signaling intersects with RTK signaling pathways, including the RAS/RAF/MEK/ERK pathway, PI3K/ΑΚΤ signaling cascade, and the JAK/STAT3 pathway^[68]^. The PI3K/ΑΚΤ signaling cascade lifts the expression of Snail and Slug via the activation of NF-κB^[74]^, while the RAS/RAF/MEK/ERK pathway phosphorylates nucleus Jun proto-oncogene, AP-1 transcription factor subunit (c-Jun) to promote the expression of EMT related transcription factors^[59,68]^. Crosstalk between the Notch signaling and TGF-β signaling via the Notch intracellular domain (NICD) and SMAD2 can also initiate EMT. After Delta-like or Jagged family binds to the Notch receptor, NICD is cleaved by γ-secretase and tumor necrosis factor-α-converting enzyme (TACE), and then translocated to the nucleus, leading by nuclear localization motif, thus activating SNAIL^[68,75]^.

Another important signaling pathway in EMT is the Wingless (WNT)/β-catenin pathway (***Fig. 4***). WNT proteins recruit Dishevelled (DVL) after binding to the membrane receptor complex of Frizzled (FZD), which stabilizes β-catenin. Glycogen synthase kinase 3 (GSK3) further determines whether β-catenin is transported into the nucleus or phosphorylated and rapidly degrades. If transported, it binds to complementary transcription factors T-cell factor (TCF)/LEF complex, as well as stimulates ZEB, SLUG, and TWIST^[76–77]^.

The expression of the serine protease inhibitor (serpin) superfamily is reported to be mainly linked with the mesenchymal subtype GBM^[78]^. Accumulating evidence has revealed an important role that serpins play in EMT. The Serpin family H member 1 (*SERPINH1*) gene, which encodes Serpin H1, better known as heat shock protein 47 (HSP47), is reported to be involved in the activation of WNT/β-catenin pathway^[79]^. In addition, HSP47 overexpression promotes ECM-related genes mainly through TGF-β signaling, which significantly contributes to tumor cell stemness and tumorigenesis^[80]^. Serpin family A member 3 (SERPINA3) expression also enhances GBM malignancy via inducing cell stemness and migration^[81–82]^. Other members of the serpin family, including SERPINE1 and SERPING1, have been proven to be positively related to GBM proliferation and progression^[78,83]^. A potential linkage between SERPINF1 or SERPINB9 and EMT has also been suggested^[84–85]^. Therefore, targeting *SERPIN* to prevent GBM invasion and recurrence might be feasible.

## Conclusions and future perspectives

GBM, a malignant brain tumor, has both high occurrence and recurrence rates. A definite diagnosis procedure starts with imaging (usually MRI) and has to undergo histopathological examinations, in that a more appropriate targeted treatment can be settled upon. Traditional treatment includes maximal surgical resection of the tumor, radiation therapy, and chemotherapy. Advanced treatments include tumor-treating fields therapy, immunotherapy, and stem cell-based therapy.

Despite all these treatment methods, current outcomes are still unsatisfactory. The medium survival time of GBM patients, ranging from 11 months to two years, have not significantly improved over the past two decades^[3,86]^. Therapy resistance and recurrence are still inevitable. Specific anti-tumor drugs can even fasten GBM cell invasion by promoting EMT, which is such a paradox that brings about frustration to patients. This phenomenon might be the result of redundant compensatory mechanisms of GBM, insufficient target coverage, or poor tolerability and safety^[87]^.

Whatsoever, efforts are made to solve the dilemma. Different signaling pathways, either associated with inbuilt gene programs or related to the microenvironment, have been discovered and elucidated in recent years. Herein, we mainly discuss the p53 pathway, RTK pathways, and EMT-related pathways, especially their roles in promoting GBM invasion. These signaling pathways offer new insights into advanced GBM therapies. New drugs are to be designed to target elements involved in these pathways.

It is undeniable that humans are still far from the destination of effective GBM treatment. Nevertheless, by exploring possible targets and methods, we can anticipate that GBM patients will have more effective treatments in the future, and thus the financial burden can be largely relieved.
